# Clinical Evaluation of the Safety and Effectiveness of *Heptonica*: A Ghanaian Hepatorestorative Polyherbal Product

**DOI:** 10.1155/2020/9596182

**Published:** 2020-06-21

**Authors:** A. O. Agyemang, M. L. K. Mensah, R. C. Yamile, A. Ocloo, A. A. Appiah, A. Y. Mensah, K. P. Thomford

**Affiliations:** ^1^Department of Herbal Medicine, Upper West Regional Hospital, Ghana Health Service, Wa, Ghana; ^2^Institute of Traditional and Alternative Medicine, University of Health and Allied Sciences, Ho, Ghana; ^3^Department of Herbal Medicine, Faculty of Pharmacy of Pharmaceutical Sciences, College of Health Sciences, Kwame Nkrumah of University and Technology, Kumasi, Ghana; ^4^Department of Medicine, Upper West Regional Hospital, Ghana Health Service, Wa, Ghana; ^5^Centre for Plant Medicine Research, Mampong-Akuapem, Ghana; ^6^Department of Biochemistry, Cell and Molecular Biology, University of Ghana, Legon, Ghana; ^7^Department of Pharmacognosy, Faculty of Pharmacy of Pharmaceutical Sciences, College of Health Sciences, Kwame Nkrumah of University and Technology, Kumasi, Ghana

## Abstract

The incidence of liver diseases is increasing globally, and many patients in developing countries are resorting to the use of herbal products as treatment. This study was aimed at establishing the safety and effectiveness outcomes for patients with deranged liver panel treated with a Ghanaian finished polyherbal product. The product *Heptonica* is prepared by CPMR from three medicinal plants: *Bidens pilosa*, *Citrus aurantifolia*, and *Trema orientalis*. Fifty (50) participants with clinical and biochemical signs of liver impairment were purposively recruited and treated for a period of 28 days. Participants received *Heptonica* at a dose of 30 mL 8 hourly after meals for the treatment period. Clinical and biochemical evaluation (liver panel test, renal function test, haematology, and urinalysis) of subjects for the safety and effectiveness of the product was undertaken at days 0 (baseline), 14, and 28. Compared to the baseline values, *Heptonica* did not have any untoward effect on renal function, haematological parameters, and urine parameters of subjects. Clinical and liver panel results of the participants also improved compared to the baseline: serum aspartate transaminase (AST) (*p* < 0.0001), alanine transaminase (ALT) (*p* < 0.0001), gamma-glutamyltransferase (GGT) (*p*- 0.0013), total bilirubin (*p*-0.0136), direct bilirubin (*p* < 0.0001), total proteins (*p*-0.0409), and alkaline phosphates (*p*- 0.0284). Level of albumin showed no significant difference within the study period. The outcome of this study indicates *Heptonica* has hepatorestorative action with no observable toxicity and can be used with confidence as indicated as a liver tonic.

## 1. Introduction

The liver is an organ responsible for many important physiological functions including metabolic, circulatory, immunological, storage, and endocrine activities in the human body. Liver (hepatic) diseases comprise a wide range of complex conditions that affect the structure and functions of the liver. Currently, about 844 million people are living with different forms of liver diseases leading to over 2 million deaths annually. Mortality from liver diseases is expected to rise across the world [1–3]. Lack of affordable and accessible therapeutic agents for the treatment of liver diseases is impeding the quality of life of patients as well as increasing mortality [4]. Ghana reported 7,833 mortalities due to liver diseases in the year 2017 that translated to 3.72% of the total annual mortality [5].

Liver diseases are mainly managed on a supportive basis, which includes the removal of causative agents and treating the underlying causes [6]. The choice of therapy depends on many factors including the type of liver disease, the stage and intensity, health status of the individual, and the availability of professionals and healthcare facilities for specific intervention services [7]. Despite the high advances in modern science, it is still a challenge to get safe and efficacious agents to improve hepatic protection and enhance hepatic functions [8].

Globally, there are limited therapeutic agents for preventing and managing liver diseases. The few therapeutic options are very expensive and not accessible to many people especially those in developing countries [9, 10]. There is a great need to search for more agents that can protect the liver and manage and treat liver diseases. Some of the few allopathic drugs for treating liver diseases are also limited in efficacy and compounded by toxic side effects [11].

Herbal products are used traditionally in the prevention, treatment of many diseases, and for improving the general wellbeing of humans including as a remedy for liver diseases and to restore normal functions of the liver [9]. However, most herbal products have not been subjected to standardization or clinical studies to prove their safety and effectiveness in humans [12]. Although there are claims of many years of using herbal medicines for liver diseases, many of such claims do not provide evidence concerning their safety and effectiveness in human clinical studies [13]. In Ghana, affordable and readily available herbal medicines are a means of treatment of liver diseases. One of such products is *Heptonica* which has been used for over 30 years at the Centre for Plant Medicine Research (CPMR) and the past seven years by medical herbalists at some of the 35 public hospitals which are pilot centres for the integration of herbal medicine into the public healthcare system in Ghana. The three plant components of *Heptonica* (*Citrus aurantifolia* (Rutaceae), *Bidens pilosa* (Compositae), and *Trema orientalis* Linn. Blume (Ulmaceae)) have ethnobotanical evidence of use in the management of liver diseases [14]. However, there has not been a comprehensive clinical study of the finished herbal product to establish its potential in therapy. The aim of this study was to provide scientific data on the safety and effectiveness of *Heptonica* for the treatment and/or management of liver diseases in humans with deranged liver panel.

## 2. Materials and Methods

### 2.1. Materials

*Heptonica* is a liquid finished herbal product produced and supplied by the Centre for Plant Medicine Research (CPMR) located at Mampong-Akuapem in the Eastern Region of Ghana. *Heptonica* is produced from the dried parts of the following: leaves of *Citrus aurantifolia* (Rutaceae), the aerial part of *Bidens pilosa* (Compositae), and the stem bark of *Trema orientalis* Linn. Blume (Ulmaceae). It is packaged in 330 mL plastic amber bottles for oral administration for adults only, at the dose of 30 mL, three times daily after meals.

### 2.2. Methods

#### 2.2.1. Study Design

An open-label prospective noncomparative clinical study was conducted among clients that patronize the Herbal Medicine Services at the Upper West Regional Hospital (UWRH). Convenience sampling technique was used to select 50 clients for the study based on the inclusion and exclusion criteria [15]. Clients with suspected liver diseases were tested for liver panel, renal function (RFT), full blood count (FBC), and urinalysis. Clients with deranged (below or above reference range) liver panel results were enrolled in the study at the Upper West Regional Hospital (UWRH). There were no positive and negative controls for the study [16, 17]. Haematological tests were done using Mindray 3000 Haematology analyzer while Mindray 3000 Biochemistry analyzer was used to run the liver and renal biochemistry tests. All tests were done according to the manufacturer's instructions. Structured questionnaires were used to obtain data such as age, sex, education level, marital status, contact number, symptoms, signs as well as a Karnofsky scale assessment for quality of life.

#### 2.2.2. Inclusion and Exclusion Criteria

The inclusion criteria for enrolment into this study comprised clients with deranged liver disease, confirmed by a biochemistry laboratory test, adults aged between 18 and 65 years, outpatients (both males and females), clinically stable subjects with no life-threatening conditions, ability to report for follow-up, and the willingness to sign consent forms.

Subjects were excluded from the study when they were below 18 years of age, critically ill clients, clients on admission, clients with a high temperature above 39°C, evidence of severe liver disease, people with comorbidities, and clients on other hepatoprotectives, recreational, or other drugs 14 days before the study. Pregnant women, breastfeeding mothers, people who are unable to come for follow-ups, and clients with any condition that might compromise any of the biochemical parameters were also excluded.

#### 2.2.3. Treatment and Follow-Ups

As mentioned above, fifty (50) participants, who met the inclusion criteria were selected for the study. Vital signs were checked, clinical assessment and laboratory tests (liver panel test, renal function test (RFT), full blood count (FBC), and urinalysis) were performed on day zero (0) for baseline data, and each subject was given two weeks supply of the herbal preparation and was asked to report for follow-ups on days 14 and 28 at the hospital.

During each follow-up, checking of vitals, clinical assessment, and laboratory tests were repeated. Home visits and phone calls were used to monitor compliance and possible adverse effects and to remind subjects of their next hospital visits. A participant was withdrawn if he/she clinically deteriorated or worsened during the study period. Any individual who developed adverse drug reaction to *Heptonica* was also withdrawn from the research. Clients were also given the opportunity to voluntarily withdraw from the study if they so wished with or without explanation to the researchers.

#### 2.2.4. Assessment of Safety

The data from clinical assessment, renal function test, haematological assessment (FBC), urinalysis, abdominal ultrasound, and adverse effects questionnaires were used to assess the safety profile of *Heptonica* in humans within the 28 days of drug administration.

#### 2.2.5. Assessment of Effectiveness

Liver panel biochemistry is an established method of assessing liver activity and it is used as a tool in assessing the integrity of the liver. The ability to significantly normalize or reverse deranged liver parameters was the primary endpoint of effectiveness with an improvement of quality of life as the secondary endpoint.

### 2.3. Ethical Considerations

Ethical approval for the study was obtained from the Institutional Review Board (Committee on Human Research, Publication and Ethics-CHRPE) at the School of Medical Sciences (SMS), Kwame Nkrumah University of Science and Technology (KNUST) and Komfo Anokye Teaching Hospital (KATH) with reference number: CHRPE/AP/038/18. Approval was also obtained from the Upper West Regional Health Directorate and the Upper West Regional Hospital, the site for the study. All participants agreed and signed informed consent forms and were assigned codes to protect their identities.

### 2.4. Statistical Analysis

GraphPad Prism version 8.02 for Windows (GraphPad Software, San Diego, CA, USA) and SPSS version 20.0 for Windows (IBM, Chicago, IL, USA) were used for all the data analysis. *p* ≤ 0.05 was considered statistically significant in all analysis.

## 3. Results and Discussion

### 3.1. Demographics and Aetiology of Participants Liver Diseases

Out of the 50 participants, 39 (78%) were males and the remaining 11 (22%) were females (Table 1). This observation is consistent with findings from other studies confirming gender differences in the prevalence of liver diseases [18]. The high rate of the deranged liver panel in males may be attributed to their risky lifestyle. Multiple sexual partners and abuse of alcohol and other drugs are more common in males than in females [19]. The data also showed that more youth and more married people had deranged liver panel compared to the other groups. Majority of the subjects were in the 20–29-year group (32%) followed by the 30–39-year group (28%) with the least being those in the 50–59-year group (6%).

Viral hepatitis (hepatitis B and C) was found to be the major cause of deranged liver panel among the participants accounting for 66% of the cases. The cause of deranged liver panel could not be ascertained for 22% (11/50) of the participants and alcoholism and hepatocellular carcinoma (HCC) were responsible for 8% (4/50) and 4% (2/50), respectively, of the cases (Table 1). Viral hepatitis has been associated with deranged liver panel in human studies, thus accounting for the majority of participants [20].

### 3.2. Safety of *Heptonica* in Human Subjects

#### 3.2.1. Effects of *Heptonica* on Vital Signs, Renal Function, and Urinalysis of the Subjects

The results of axillary temperature, body weight, and blood pressure of participants at the baseline (day 0) and end of the study (day 28) were as presented in Table 2. All the parameters at the baseline were within the recommended reference range for adults, and although 8% of the participants complained about fever, the axillary temperatures were within the normal range.

The administration of *Heptonica* had no significant effect on the axillary temperature, body weight, and blood pressure of the participants over the 28-day period of the study compared to the baseline values except for the slight reduction in the diastolic blood pressure (*p* value = 0.0496). The slight reduction in the diastolic blood pressure was within the recommended adult diastolic blood pressure reference range and therefore not a health risk (Table 2).

The biochemical parameters that were assessed in the participants as a measure of renal function were urea, creatinine, blood urea nitrogen (BUN), and electrolytes (sodium, potassium, and chloride) as shown in Table 2. The presence of one or more deranged biochemistry levels indicates renal injury [21]. The results show that all the parameters were within the reference range on day 0 (baseline) and that administration of the *Heptonica* did not result in significant changes in any of the parameters except for chloride, where the values on day 28 were significantly higher compared to the baseline (day 0). However, the chloride levels on day 28 were still within the reference range for adults and thus may not pose any health risk.

It has been established in animal studies that *Bidens pilosa* improves the structure and function of the renal system by enhancing the regeneration of the renal tubules and protects renal tissues exposed to carcinogens [22]. *Citrus aurantifolia* have been proven to be nephroprotective in animal studies by preventing stress-related renal damage [23, 24]. The use of the aerial parts of *Trema orientalis* in animal studies did not provide any evidence of renal injury in animal toxicity studies and so it is expected not to cause renal damage in humans [25].

Furthermore, assessment of urinalysis in the participants revealed a slight rise in urine pH (*p* value = 0.049) (Table 2). Human studies have confirmed that the use of *Citrus orientalis* results in the alkalinisation of urine by increasing urine pH [26]. Average pus cell concentrations reduced slightly from 2.84 (0.53) to 1.86 (0.18) (*p* value = 0.050) (Table 2). *B. pilosa*, *C. aurantifolia*, and *T. orientalis* have antibacterial properties giving *Heptonica* ability to reduce pus cells [27–29].

#### 3.2.2. Effects of *Heptonica* on Quality of Urine

The results of urine quality assessment on days 0 and 28 are shown in Table 3. The *Heptonica* was able to improve the urine appearance from hazy to clear. All 2 (4%) of the participants who had blood in urine and positive urine bilirubin got cleared by the 28^th^ day. *Bidens pilosa* has proven diuretic and choleretic (improving bile flow) properties [30]. Once a product can improve the normal functioning of the liver, it can now conjugate bilirubin and transport it actively into bile as a choleretic and for excretion, thereby reducing plasma bilirubin concentration [31]. Calcium oxalate crystals in the urine as well as the 2% of urine leucocytes were all reduced within the 28-day period. This result agrees with those from other human studies where oral administration of *Citrus aurantifolia* resulted in the decrease of urine calcium ratio in people with idiopathic calcium renal calculi [26].

#### 3.2.3. Effect of *Heptonica* on Haematological Parameters

The haematological test results (Table 4) showed that all the parameters were within the normal reference range for an African adult on day 0 (baseline) [32]. Administration of *Heptonica* led to significant increases in haemoglobin level, MCV, MCH, and MCHC levels at day 28 compared to the baseline values (Table 4). The decoction of the bark of *Trema orientalis*, one of the components of *Heptonica*, is used in treating anaemia as a haematinic and this may account for its ability to improve haemoglobin [33]. *Trema orientalis* has been proven in animal studies as haematoprotective and a haematopoietic agent that improves haemoglobin, MCH, and MCV [34].

### 3.3. Reported Adverse Effects of *Heptonica*

Out of the 50 participants who were enrolled in this study, two (2) males (4%) reported side effects after taking *Heptonica*. The first client experienced mild diarrhoea for the first two days, but this was resolved when treatment continued without the administration of any other agent. The second client reported itching and macular rashes on the left elbow which stopped after 24 hours on continuing taking the *Heptonica*.

### 3.4. Effectiveness of *Heptonica* in Human Subjects

#### 3.4.1. Effect of *Heptonica* on Reported Clinical Symptoms

Participants reported different symptoms presumably associated with their liver diseases. Symptomatic evaluations of participants were done on days 0 and 28. The recorded symptoms were based on the compilation and frequency of all the complaints of the 50 participants.

**Figure 1:**
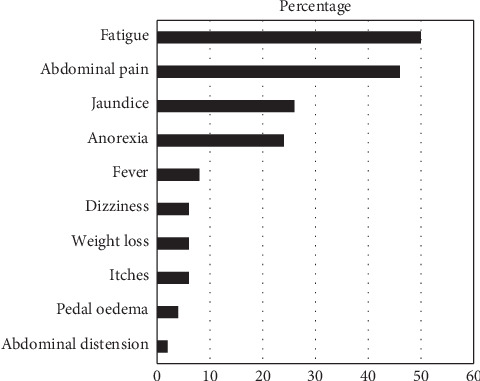
Symptoms of liver disease as stated by participants on day 0.

As shown in Figure 1, the participants enrolled in the study had ten common presenting complaints among which fatigue and abdominal pains were the most common complaints while abdominal distension was the least. Abdominal ultrasound confirmed the abdominal distension cases as ascites secondary to liver disease. On day 0, fatigue, abdominal pain, and jaundice were the highest numbers of symptoms complained about in descending order and abdominal distension was the least complaint. On 14^th^ day review, almost all the symptoms complained by the participants showed some level of improvement (data not shown), and further improvement was observed on day 28 (Table 5). Thus, after 28 days of taking *Heptonica*, most clients experienced maximum relief of symptoms compared with their presenting complaints on day 0. Jaundice and fever conditions showed the highest recovery of 75% each while ascites had the lowest change of 100% mild improvement. These observations imply that the *Heptonica* appears to be improving the quality of life of the participants.

*Heptonica* had relieved most of the clients' symptoms especially fever and jaundice but showed less effect on ascites. Total proteins improved significantly but not albumin, implying that the increase came from other proteins other than albumin. The relief of symptoms of participants follows the pattern of improvement seen from the laboratory results and could be suggestive of possible modes of action of *Heptonica*. By clearing jaundice, *Heptonica* may be acting as a choleretic reducing hyperbilirubinemia responsible for the eyes yellow coloration and pruritus. *Heptonica* also possess antipyretic activity because it relieved the participants of fever. *Bidens pilosa* and *Citrus aurantifolia* have been proven to be antipyretics [35].

#### 3.4.2. Effect of *Heptonica* on Liver Panel of Participants

All participants were assessed for AST, ALT, GGT, bilirubin (total and direct), proteins (total and albumin), and alkaline phosphatase (ALP) on days 0, 14, and 28.  Table 6 shows the levels of these parameters on days 0 and 28. Each client enrolled in the study had some form of deranged liver panel on day 0 (baseline). *R*-value which is a ratio of alanine transaminase to alkaline phosphatase (ALT/ALP) is used as an indicator to predict the possible types of liver injuries. At baseline, it was observed that the majority (90%) of the participants had a cholestatic liver disease while the remaining 10% had a cholestatic-hepatocellular disease. There was a significant improvement in the AST, ALT, GGT, bilirubin (total and direct), and ALP after 28 days of treatment compared to the baseline results, suggesting that the herbal preparation (*Heptonica*) improves liver biochemistry and may be effective in the management of liver diseases. The clearance of jaundice in the eyes of all the 26% of the participants who were icteric after two weeks of treatment. These patients had jaundice secondary to hepatic causes, and its clearance gives credence to the activity of the intervention product, *Heptonica*, in the management of liver diseases.

Therapeutic agents are administered in liver diseases to halt the progression of the disease, stimulate and improve hepatocyte integrity and regeneration capacity and liver function, reduce complications as well as treating the underlying cause of the disease, and improve the quality of life of the patient [36]. *Heptonica* acted as an anti-inflammatory agent by significantly reducing the AST and ALT which are produced in response to liver inflammation (Table 6). *Bidens pilosa*, *Citrus aurantifolia*, and *Trema orientalis* as components of *Heptonica* have been proven individually to possess anti-inflammatory properties [37–39]. These plants may act individually or in combination on the human liver to reduce hepatitis. *Citrus aurantifolia* can induce hepatoprotective enzymes, block genetic material damage in humans, and reverse cholestatic liver fibrosis caused by bile duct ligation [40, 41]. These may also be part of the reasons why *Heptonica* improved the liver panel of participants which were deranged.

#### 3.4.3. Effect of *Heptonica* on Different Aetiologies of Hepatic Impairment

Cholestatic injury is defined as a disproportionate elevation of ALP level as compared with AST and ALT levels. *Heptonica* significantly improved AST, ALT, GGT, and total and direct bilirubin (Table 6) in participants with cholestatic liver disease. *Heptonica* may be responsible for the anti-inflammatory effect on the liver by reducing inflammatory enzymes AST and ALT. *B pilosa* and *C. aurantifolia* which are stated components of *Heptonica* have been established to possess anti-inflammatory activity on the liver by reversing hepatic fibrosis and blocking genetic damage to hepatocytes [42–44].

**Figure 2:**
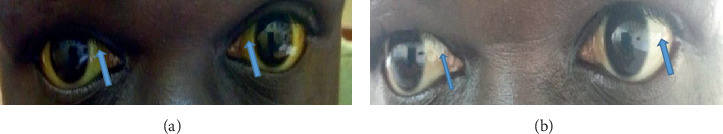
Participant with jaundiced eyes (a) and after 2 weeks of treatment (b).

Accumulation of serum bilirubin levels above 3 mg/dl leads to jaundice (Figure 2). The excretory functions of the liver were enhanced significantly by the reduction of the bilirubin (total and direct). A previous animal study proved that *Citrus aurantifolia* which is a component of *Heptonica* can reverse cholestatic liver fibrosis caused by the ligation of the bile duct [41]. *Heptonica* by reducing direct and total bilirubin may possess choleretic activity by causing more bile secretion or improving bile flow through the hepatic ducts. *B. pilosa* demonstrated protection of the liver against cholestatic liver disease in animal studies [45]. Demonstration of the clearance of jaundice from the eyes has been shown in Figure 2.

It was observed that participants with cholestatic liver disease experienced better improvement compared to those with a cholestatic-hepatocellular disease (Table 6). It is possible *Heptonica* may have improved the hepatocytes function, increased bile formation, and improved bile flow through the hepatic ducts. *B. pilosa* has been reported to normalize bilirubin concentration in blood [30].

Also, there was a significant improvement in the liver panel of participants with viral hepatitis with respect to AST, ALT, GGT, and total and direct bilirubin comparing the end of 28 days with the baseline (Table 7). It is a possibility that *Heptonica* significantly improved participants' deranged liver panel by viral hepatitis due to *Heptonica's* antiviral properties. *Heptonica* may have achieved the observed improved liver condition as an adaptogen stimulating and promoting hepatocytes regeneration and improving hepatocyte integrity [46].

Moreover, there were no significant differences between the baseline and the end of the 28-day study period in participants with deranged liver state of unknown origin. Details of the results are presented in Table 7. There was no indication that *Heptonica* could manage the actual cause of the derangement of these participants. Furthermore, there were no significant differences between the results of the baseline and the 28-day period in participants with alcoholic liver disease (Table 7). It is possible that *Heptonica* does not have much influence on the liver biochemistry of alcohol-induced liver diseases.

#### 3.4.4. Effect of *Heptonica* on Quality of Life of the Participants

On day 0, the mean score of 80 ± 0.4 Karnofsky scale of the participants improved significantly by day 28 to 91.40 ± 1.21 (*p* value < 0.0001). The improvement of quality of life may be from other activities of the product that were not investigated in this study. Some of the 94% of participants that had improved quality of life stated that they had better stamina, improved appetite, quality sleep, and general health benefits after taking *Heptonica*. Three clients (6%) who were not responding to treatment on day 28 were referred to the allopathic section of the Upper West Regional Hospital for further management. The first was a case of hepatocellular carcinoma and the other two were on account of nonresolving ascites secondary to viral hepatitis. The treatment outcomes of participants demonstrated that *Heptonica* improved clients' quality of life.

## 4. Conclusion

Clinical evaluation of *Heptonica* on urinalysis, renal function, and haematological parameters within the 28-day period of administration showed evidence of safety and effectiveness in use in the management of some liver diseases in humans. On effectiveness, *Heptonica* improved significantly AST, ALT, GGT, and bilirubin (total and direct) levels of the 50 participants which were all deranged on day 0 before the product administration. AST, ALT, GGT, total bilirubin, direct bilirubin, and alkaline phosphatase all had significant improvement compared to the baseline more especially in viral hepatitis and cholestatic liver diseases. The study has demonstrated that *Heptonica* has an effective hepatorestorative action with no observable toxicity and can be used with confidence as indicated as a liver tonic.

## Figures and Tables

**Table 1:** Demographics and aetiology of liver diseases of participants.

Parameters	Age (years)						Total
	<19	20–29	30–39	40–49	50–59	>60	
Gender							
Male	4	11	14	3	2	5	39 (78%)
Female	0	5	0	3	1	2	11 (22%)
Marital status							
Single	4	11	3	1	1	0	20 (40%)
Married	0	5	11	4	1	6	27 (54%)
Widow	0	0	0	1	1	1	3 (6%)
Condition							
Hepatitis B	3	14	8	1	0	3	29 (58%)
Hepatitis C	0	0	2	0	2	0	4 (8%)
Alcoholic liver disease	0	0	1	1	0	2	4 (8%)
Unknown cause	1	2	2	3	1	2	11 (22%)
Liver cancer	0	0	1	1	0	0	2 (4%)

**Table 2:** Safety profile of *Heptonica* on vital signs, renal function, and quantitative urine parameters.

Parameter	Day 0	Day 28	*p* value
Vital signs (*n*, 50)			
Temperature^†^ (°C)	36.10 ± 0.65	36.00 ± 0.5	0.438
Weight (kg)	60.50 (12.25)	61.00 ± 11.25	0.679
Systolic BP^†^ (mmHg)	110.0 ± 25.50	110.0 ± 20.00	0.149
Diastolic BP (mmHg)	70.00 (10.00)	70.00 ± 0.00	0.050
Renal function (*n*, 50)			
Sodium^†^ (mmol/l)	137.7 ± 6.3	136.6 ± 8.3	0.248
Potassium (mmol/l)	3.9 (0.4)	4.1 ± 0.6	0.478
Chloride (*μ*mol/l)	104.0 (3.2)	106.1 ± 3.9	**0.001**
Urea (mmol/l)	5.4 (1.8)	5.45 ± 2.1	0.829
Creatinine (*μ*mol/l)	79.4 (18.5)	81.5 ± 21.0	0.613
BUN (mg/dl)	15.7 (6.4)	15.2 ± 4.3	0.607
Urine (*n*, 50)			
pH	6.48 ± 0.13	6.82 ± 0.14	**0.049**
Specific gravity^†^	1.02 ± <0.001	1.02 ± <0.001	0.414
Pus cell	2.84 (0.53)	1.86 ± 0.18	0.050
RBC	0.12 (0.08)	0.1 ± 0.08	0.688
Epithelial cells	2.76 (0.28)	2.74 ± 0.24	0.363

Data are presented as median (interquartile range (IQR)) (Wilcoxon matched-pairs signed-rank test); “†” indicates that data were parametric and were compared using paired *t*-test. *p* ≤ 0.05 was considered significant when compared to baseline.

**Table 3:** Effect of *Heptonica* on qualitative urine parameters (*n*, 50).

Parameter	Details	Day 0	Day 14	Day 28
		%	%	%
Urine colour	Straw	98	84	90
	Amber	2	16	10
Urine appearance	Clear	68	68	78
	Hazy	26	18	16
	Cloudy	6	14	6
Urine proteins	Negative	88	84	90
	Trace	8	14	6
	Positive	4	2	4
Urine glucose	Negative	100	100	100
Urine ketones	Negative	100	100	100
Urine blood	Negative	96	98	100
	Trace	2	2	0
	Positive	2	0	0
Urine bilirubin	Negative	96	100	100
	Positive	4	0	0
Leucocytes	Negative	78	84	96
	Trace	10	8	4
	Positive	12	8	0
Urobilinogen	Normal	92	98	98
	Increased	8	2	2
Cast	Not seen	100	100	96
	Seen	0	0	4
Crystals	Not seen	96	98	98
	Calcium oxalate	4	2	2

**Table 4:** Assessment of *Heptonica* on haematological parameters.

Haematology	Day 0	Day 28	*p* value
WBC	5.2 (0.2)	5.1 (0.2)	0.191
Lymph #^†^	2.1 ± 0.1	2.1 ± 0.1	0.531
Mid #	0.5 (0.0)	0.45 (0.0)	0.156
Gran #	2.8 (0.2)	2.8 (0.2)	0.990
Lymph %	39.6 (1.5)	41.3 (1.5)	0.274
Mid %	9.6 (0.3)	8.8 (0.3)	0.080
Gran %^†^	49.4 ± 1.3	50.3 ± 1.5	0.547
Hb^†^	13.3 ± 0.3	13.7 ± 0.3	**0.002**
RBC^†^	4.7 ± 0.1	4.6 ± 0.1	0.569
MCV	85.9 (1.2)	87.6 (0.9)	**0.029**
MCH	29.4 (0.6)	30.8 (0.4)	**<0.0001**
MCHC	33.4 (0.3)	34.0 (0.4)	**0.002**
PLT^†^	224.6 ± 11.5	231.3 ± 12.2	0.427

Data are presented as median (interquartile range (IQR)) (Wilcoxon matched-pairs signed-rank test); “†” indicates that data were parametric and were compared using paired *t*-test. *p* ≤ 0.05 was considered significant when compared to baseline. WBC: white blood cells; lymph: lymphocytes; mid cells: monocytes; granulocytes: basophils, eosinophils, and neutrophils; RBC: red blood cells; Hb: haemoglobin; MCV: mean corpuscular volume; MCH: mean corpuscular haemoglobin; MCHC: mean corpuscular haemoglobin concentration; PLT: platelet.

**Table 5:** Assessed symptoms of liver disease on day 28.

Symptoms	No improvement (%)	Mild improvement (%)	Moderate improvement (%)	Maximum improvement (%)
Jaundice	0.00	0.00	25.00	75.00
Abdominal pain	0.00	20.83	33.33	45.83
Fatigue	0.00	5.26	36.84	57.89
Anorexia	0.00	0.00	40.00	60.00
Abdominal distension	0.00	100.00	0.00	0.00
Pedal oedema	0.00	0.00	100.00	0.00
Itches	0.00	0.00	33.33	66.67
Weight loss	0.00	50.00	0.00	50.00
Fever	0.00	0.00	25.00	75.00
Dizziness	0.00	0.00	50.00	50.00

**Table 6:** *Heptonica* on types of liver diseases.

Liver parameters	Day 0	Day 28	*p* value
General participants (*n*, 50)			
AST (U/l)	40.5 (46.5)	27.8 (19.4)	<**0.0001**
ALT (U/l)	44.0 (45.6)	25.8 (65)	<**0.0001**
GGT (U/l)	58.6 (119.5)	40.1 (101.6)	**0.0013**
Total bilirubin (*μ*mol/l)	25.9 (38.2)	15.2 (17.6)	**0.0136**
Direct bilirubin (*μ*mol/l)	7.6 (13.5)	3.8 (2.9)	**<0.0001**
Total proteins^†^ (g/dl)	70.8 ± 10.5	74.8 ± 9.3	**0.0409**
Albumin^†^ (g/dl)	42.0 ± 7.3	42.0 ± 6.0	0.3432
ALP (U/l)	176.9 (175.6)	171.4 (153.5)	**0.0284**
Cholestatic (*n*, 45)			
AST (U/l)	41.00 (47.15)	28.00 (19.25)	**0.0004**
ALT (U/l)	43.00 (47.10)	25.00 (27.90)	**0.0004**
GGT (U/l)	47.30 (106.30)	37.00 (51.05)	**0.0084**
Total bilirubin (*μ*mol/l)	27.80 (48.30)	16.40 (19.90)	**0.0101**
Direct bilirubin (*μ*mol/l)	8.00 (12.70)	3.70 (3.00)	<**0.0001**
Total proteins^†^ (g/dl)	70.80 ± 12.00	75.00 ± 9.50	0.0928
Albumin^†^ (g/dl)	42.00 ± 7.00	41.00 ± 5.40	0.3027
ALP (U/l)	192.40 (161.90)	176.20 (142.70)	**0.0322**
Hepatocellular-cholestatic (*n*, 5)			
AST (U/l)	49.02 (13.80)	30.98 (8.75)	0.0625
ALT (U/l)	46.36 (5.81)	31.56 (6.50)	0.0625
GGT (U/l)	213.30 (42.84)	161.70 (49.99)	0.0625
Total bilirubin (*μ*mol/l)	11.04 (3.80)	11.02 (0.56)	0.8125
Direct bilirubin (*μ*mol/l)	6.56 (2.29)	4.82 (0.94)	0.8125
Total proteins^†^ (g/dl)	65.68 ± 5.35	74.00 ± 2.92	0.3125
Albumin^†^ (g/dl)	43.24 ± 2.37	43.40 ± 1.29	>0.9999
ALP (U/l)	14.08 (1.40)	13.22 (1.30)	>0.9999

Data are presented as median (interquartile range (IQR)) (Wilcoxon matched-pairs signed-rank test); “†” indicates that data were parametric and were compared using paired *t*-test. *p* ≤ 0.05 was considered significant when compared to baseline. AST: aspartate aminotransferase; ALT: alanine aminotransferase; GGT: gamma-glutamyltransferase; ALP: alkaline phosphatase.

**Table 7:** *Heptonica* on different aetiologies of liver impairment.

	Day 0	Day 28	*p* value
Viral hepatitis (*n*, 33)			
AST (U/l)	38.9 (42.40)	29.3 (13.8)	**0.0001**
ALT (U/l)	47.0 (44.75)	33.8 (31.5)	**0.0014**
GGT (U/l)	55.0 (121.50)	39.2 (101.45)	**0.0238**
Total bilirubin (*μ*mol/l)	27.9 (39.45)	18.9 (21.6)	**0.0353**
Direct bilirubin (*μ*mol/l)	8.0 (15.65)	3.7 ± (3.0)	**0.0005**
Total proteins^†^ (g/dl)	70.8 ± 13.25	74 ± 11.0	0.2878
Albumin^†^ (g/dl)	42.96 ± 8.20	42 ± 5.5	0.3348
ALP (U/l)	204.4 (167.8)	176.2 (133.3)	**0.0163**
Alcohol-induced liver disease (*n*, 4)			
AST (U/l)	87.9 (20.7)	49.0 (16.2)	0.3750
ALT (U/l)	87.1 (17.2)	44.7 (8.8)	0.1250
GGT (U/l)	250.6 (155.3)	107.5 (59.1)	0.1250
Total bilirubin (*μ*mol/l)	18.4 (11.3)	10.7 (2.1)	0.6250
Direct bilirubin (*μ*mol/l)	6.5 (3.3)	5.7 (0.9)	>0.9999
Total proteins^†^ (g/dl)	71.5 ± 5.9	70.5 ± 1.8	0.8750
Albumin^†^ (g/dl)	37.25 ± 2.1	39.00 ± 0.7	0.6250
ALP (U/l)	145.2 (40.2)	78.00 (26.1)	0.1250
Unknown (*n*, 11)			
AST (U/l)	25.5 (27.0)	22 (10.6)	0.1472
ALT (U/l)	22.6 (29.4)	19.7 (5.0)	0.0674
GGT (U/l)	24 (114.3)	23.4 (24.0)	0.1016
Total bilirubin (*μ*mol/l)	21.9 (19.7)	11.9 (9.8)	0.1426
Direct bilirubin (*μ*mol/l)	7.8 (7.1)	3.7 (3.2)	0.0693
Total proteins^†^ (g/dl)	66 ± 18.4	75 ± 9.0	0.1406
Albumin^†^ (g/dl)	44.8 ± 7.0	43 ± 5.0	0.5155
ALP (U/l)	128.6 (121.3)	135 (186.9)	0.8135

Data are presented as median (interquartile range (IQR)) (Wilcoxon matched-pairs signed-rank test); “†” indicates that data were parametric and were compared using paired *t*-test. *p* ≤ 0.05 was considered significant when compared to baseline. AST: aspartate aminotransferase; ALT: alanine aminotransferase; GGT: gamma-glutamyltransferase; ALP: alkaline phosphatase.

## Data Availability

Raw data will be made available upon request to the corresponding author.
